# Effect of Filler Wire Composition on Weld Metal Microstructure and Mechanical Properties in X80 Steel Laser Welds

**DOI:** 10.3390/ma17215235

**Published:** 2024-10-28

**Authors:** Hanwen Yang, James Chen, Xiaoye Zhao, Nazmul Huda, Adrian P. Gerlich

**Affiliations:** 1 Centre for Advanced Materials Joining (CAMJ), Department of Mechanical and Mechatronic Engineering, University of Waterloo, 200 University Avenue West, Waterloo, ON N2L 3G1, Canada; x342zhao@uwaterloo.ca (X.Z.); n2huda@uwaterloo.ca (N.H.); gerlich@uwaterloo.ca (A.P.G.); 2 CanmetMATERIALS, Natural Resources Canada, 183 Longwood Road South, Hamilton, ON L8P 0A5, Canada; jameszheng.chen@nrcan-rncan.gc.ca

**Keywords:** laser, welding, pipeline, microstructure, microhardness

## Abstract

Laser welding was performed using different filler wires, ER70S steel, commercially pure iron, and pure nickel filler, in the context of welding X80 pipeline steel to assess the microstructure and mechanical properties of the weld metal. Introducing an ER70S wire promoted acicular ferrite formation in the fusion zone, compared to a bainitic microstructure in an autogenous laser weld. The use of pure iron wire was considered as a potential strategy for reducing hardenability, as it led to the dilution of alloying elements in the fusion zone, increasing ferrite content and reducing weld metal hardness to a level compliant with API pipeline standards. The addition of pure nickel wire was used to reveal the degree of weld metal mixing imposed by the laser (thus providing an unambiguous tracer element) when it is combined with filler material dilution in the fusion zone, revealing that the upper region contained 38% wire material and the lower region only 12%. This accounts for the differences observed between the upper versus lower portions of the weld metal when other wires are used, and the use of hardness mapping and micro-indentation demonstrates the correlation between the variations in mechanical properties and microstructural differences introduced by incomplete mixing of the filler wire elements.

## 1. Introduction

Micro-alloyed high-strength low-alloy (HSLA) steels are extensively employed in a variety of structural applications, including oil and gas pipelines, shipbuilding, and bridge construction, due to their optimal balance of enhanced mechanical performance and economic viability [1]. They are produced using thermo-mechanically controlled processing (TMCP), a technique that refines the microstructure, resulting in a material that exhibits both high strength and exceptional toughness. During welding, the weld metal can only experience cooling at various rates, and thus relies instead on a high degree of alloying to achieve strength overmatching with HSLA base metals. However, care must be taken with the selection of alloying additions, as welding consumables with a high carbon equivalent will increase the sensitivity to cold cracking and could deteriorate low-temperature toughness, especially if high cooling rates are imposed.

Over conventional arc welding methods, laser welding leads to a high welding speed, low heat input and small heat affected zone (HAZ), which make it a promising technique in pipeline welding, especially for the root pass. The use of laser welding of pipe materials has been implemented in industry for more than 20 years, in cases where there is an emphasis for higher productivity [2,3]. The high depth to width ratio of the laser weld can help reduce the total number of weld passes required to fill the groove, which significantly increases the productivity. 

Using a laser as the primary heat source, welding of thick materials can be readily achieved due to the deep penetration when a keyhole is formed. Autogenous laser welding of thick carbon steel presents certain technical difficulties, notably a reduced capability for bridging gaps effectively, especially when using a fine laser spot size (<1 mm), and a high cooling rate, which may promote formation of hard and brittle microstructures in the fusion zone (FZ). These limitations can be overcome by adding a filler wire into the weld molten pool. However, the majority of commercial wires are developed and optimized for conventional arc welding methods. In the case of narrow-gap joints, the welds are particularly sensitive to the higher carbon equivalent since this will promote higher weld metal hardness, which is shown in a previous work [4]. The deep, narrow weld profile of the laser weld and the resulting high cooling rate would result in a high hardness that exceeds the allowed value in the API 5L pipeline industry standard [5]. In our previous study, introducing an ER70S steel wire during laser welding of X80 pipeline steel promoted the formation of acicular ferrite (AF) and bainite (B) in the fusion zone, with a higher hardness than that required by API standard for pipes used in sour gas applications (not to exceed 275 HV) [4,6]. 

The deep and narrow FZ geometry of a laser weld usually leads to an uneven distribution in filler metal alloy elements along the through-thickness direction. It has been shown that a nickel-based wire can be used to trace the filler material distribution during hybrid laser-arc welding of a 20 mm thick steel [7], and the homogeneity and dilution of the filler wire were revealed utilizing energy-dispersive X-ray spectroscopy (EDS). Nickel can promote solid solution hardening of ferrite, and influence the microstructure by suppressing the γ to α transformation [8]. Mao et al. [9] found that 2% to 6% Ni favored the formation of more acicular ferrite (AF) and lath martensite (LM), at the expense of polygonal ferrite (PF). Notably, at the higher end of this nickel concentration range (6%), the weld metal exhibited a significant proportion of martensite, reaching up to 75% [9].

In this work, a pure iron wire was fed during laser root welding of X80 steel to dilute the alloying elements from the base metal plate in the fusion zone. A pure nickel wire was also used to help indicate the dilution of filler material in the fusion zone. These filler wires were also compared to an autogenous and ER70S-wire-fed laser weld to provide a comprehensive comparison of the role of FZ chemistry. The influence of wire chemistry on weld metal microstructural evolution and local mechanical properties was studied, and the process–structure–property relationships are discussed in detail.

## 2. Experimental Section

**Figure 1:**
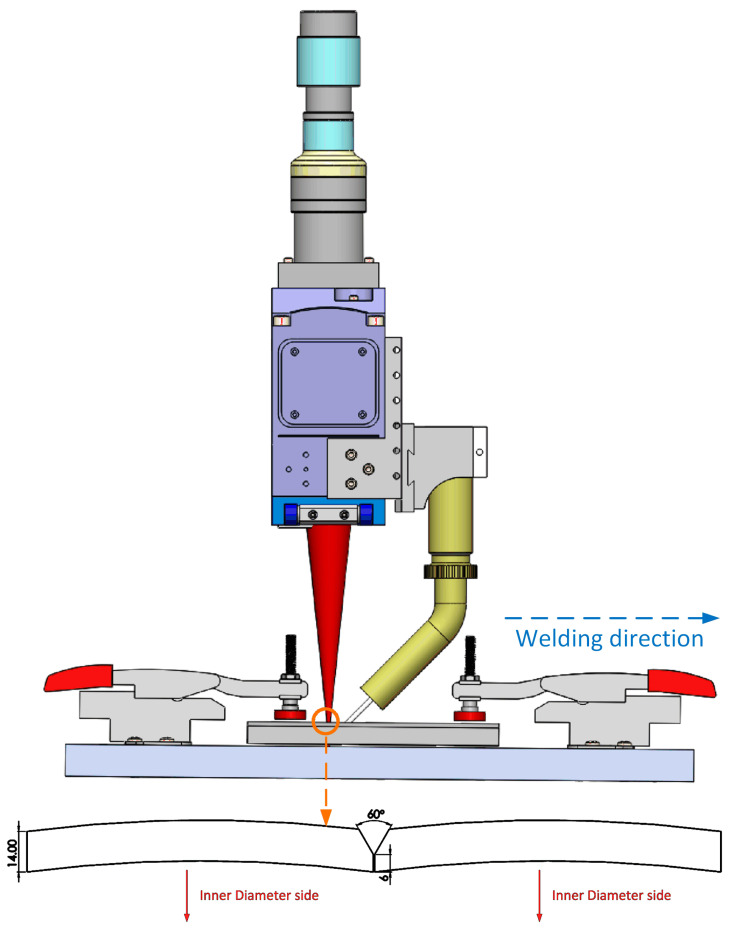
Experimental setup for the laser welding process with the Y-shaped groove configuration, similar to the setup used in a previous report [6].

The base metal employed in this work consisted of X80 pipeline steel plates, measuring 250 × 120 × 14 mm, with an inner diameter of 940 mm. The chemical composition and the calculated carbon equivalent *P_cm_* of the base material are detailed in Table 1. A ‘Y’-shaped groove geometry, featuring a 30° bevel, a 6 mm root height, and a 0.4 mm root gap, was implemented (Figure 1). All welding wires utilized in this work had the same diameter of 0.9 mm. The chemistry and *P_cm_* of ER70S-6 wire are presented in Table 2. The iron wire had a purity of 99.95%, while the nickel wire had a purity of 99%.

An IPG YLS-8000 laser system, capable of delivering an 8 kW output power and featuring a spot diameter of 1.2 mm, was employed in the study. All welds were produced utilizing a laser power of 8 kW and a travel speed of 1 m/min. The welding wire was fed in front of the laser beam, with a wire feed rate of 6 m/min. The laser spot was defocused 3 mm below the top of the groove to maximize the penetration. Argon was utilized as shielding gas, maintained at a flow rate of 18 L per min. A comprehensive summary of the welding variables is provided in Table 3. Preheating was not applied to facilitate a higher production rate.

Following welding, cross-sectional samples were extracted, polished and then etched with a Nital solution (5% nitric acid, 95% ethanol). Macrographs were captured using a stereoscope, and a scanning electron microscope (SEM) was employed for detailed microstructure characterization of the weld metal. The volume fraction of microconstituents was determined using the point counting method as outlined in ASTM E562, employing a point array containing 100 points. The reported fraction represents the average of counting results from 5 micrographs taken at various locations, presenting a 95% confidence interval (CI). EDS mapping was carried out in subzones of the FZ with a 1.2 mm × 0.8 mm area and a collection time of 5 min, and the elemental contents of Mn, Si and Ni were reported due to their relatively high fractions. EDS line-scanning, with a step size of 20 μm, was conducted across the through-thickness direction to analyze the distribution of nickel within the nickel wire weld. 

Vickers microhardness measurements across the weld area, spanning from the weld centerline to the base material, were performed using a tester provided by Clemex Technologies (Brossard, QC, Canada). Microhardness measurements were conducted using a grid pattern with indentations spaced 200 μm apart horizontally and 250 μm apart vertically. Each indentation was made using a 500-gf load applied for a dwell time of 10 s. The through-thickness hardness distribution within the fusion zone was evaluated by calculating the average hardness values obtained from four indentation lines positioned within the FZ. To determine the average hardness at various locations across the FZ, a minimum of 40 indentations were averaged, and the associated 95% CIs are reported for statistical analysis. An instrumented micro-indenter provided by NANOVEA (Irvine, CA, USA) was utilized to evaluate the local yield strength of the welds, and the technology was previously validated in several report [10]. A flat-tipped indenter with a diameter of 50 microns was used, applying a peak load of 8 N at a rate of 16 N/min. Five indentations were performed at each location, and the average yield strength was presented based on a 95% CI.

## 3. Results and Discussion

### 3.1. Weld Morphologies

**Figure 2:**
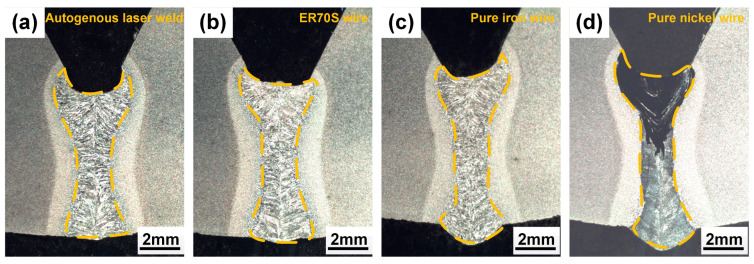
Cross-sectional morphologies: (**a**) autogenous laser weld; (**b**) laser weld with ER70S wire; (**c**) laser weld with pure iron wire; (**d**) laser weld with nickel wire.

**Figure 3:**
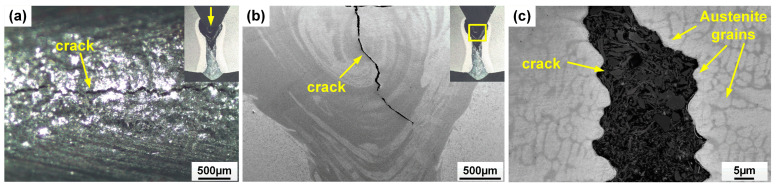
(**a**) Top surface of the laser weld with pure nickel wire, showing the presence of cracks; (**b**,**c**) SEM images of cross-sectional morphology of the laser weld with pure nickel wire, showing the presence of cracks in the fusion zone.

The morphologies of weld cross-sections are shown in Figure 2. Deep and narrow laser welds were obtained, and full penetration was achieved. Figure 3 provides a clearer view of the weld with Ni wire, showing the presence of fine centerline crack in the fusion zone. The high Ni content (>25%) could lead to a fully austenite microstructure based on the Schaeffler diagram [11]. And the austenite microstructure is sensitive to hot cracking resulting from a low thermal conductivity and high thermal expansion coefficient, which increase thermal stresses during welding, and the reduced solubility of impurities like sulfur, phosphorus, silicon, and boron in austenite, leading to their segregation at grain boundaries and the formation of low-melting-point eutectics liquid [12]. The rapid solidification characteristic of laser welding, coupled with the associated shrinkage strains, can cause the rupture of intergranular liquid films and initiate cracks as the weld solidifies [13,14].

### 3.2. Elemental Distribution and Microstructure

**Figure 4:**
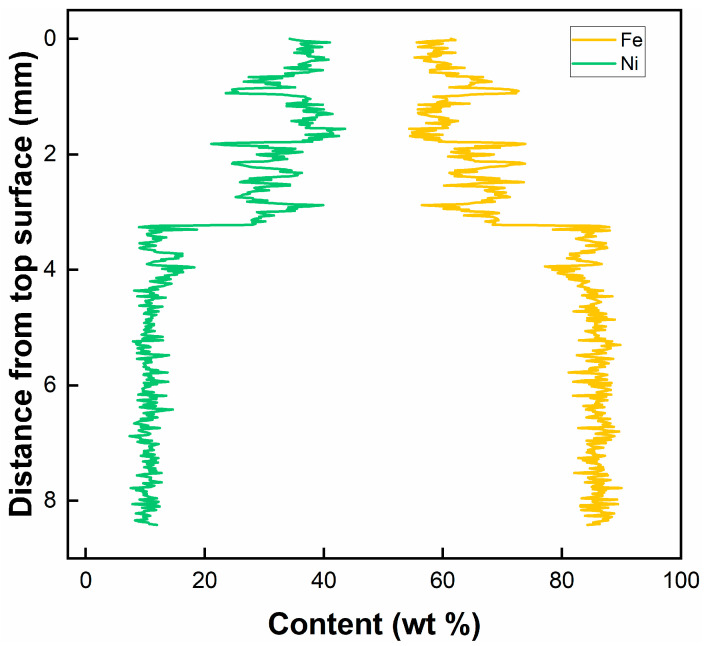
EDS line-scan results showing the distribution of Ni and Fe in the laser weld with pure nickel wire.

Table 4 summarizes the chemical composition of the key tracer elements obtained through EDS. Feeding ER70S wire increases the silicon concentration in the weld metal, reflecting the higher silicon fraction in the filler wire. The addition of pure iron wire resulted in a noticeable dilution of alloying elements within the fusion zone, as the manganese content decreased from 1.72–1.79 wt.% to 1.24–1.30 wt.%, compared to the autogenous laser weld. The distribution of nickel across the thickness of the laser weld with pure nickel wire, as shown in Figure 4, demonstrates the filler material mixing along the depth of the laser weld. A higher Ni fraction was found in the upper portion of the FZ, compared to the lower region, indicating an uneven distribution of the filler metal. The upper and lower portion of the fusion zone contained around 38% and 12% of the filler material, respectively. The dilution rates may vary for other steel wires due to different melting points, viscosity, and surface tension, but they provided a guideline for estimating and understanding dilution during laser welding. 

**Figure 5:**
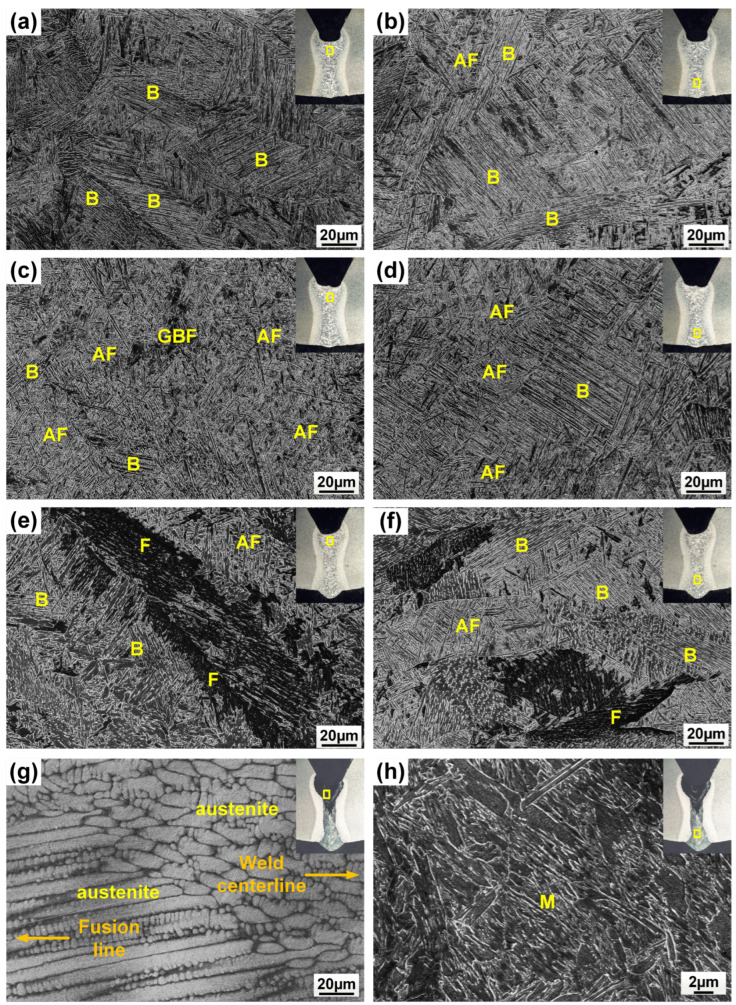
SEM images showing the presence of ferrite (F), acicular ferrite (AF), grain boundary ferrite (GBF), bainite (B) and martensite (M) in different regions of the weld metal: (**a**,**b**) autogenous laser weld; (**c**,**d**) the weld with ER70S wire; (**e**,**f**) the weld with pure iron wire; (**g**,**h**) the weld with pure nickel wire.

**Figure 6:**
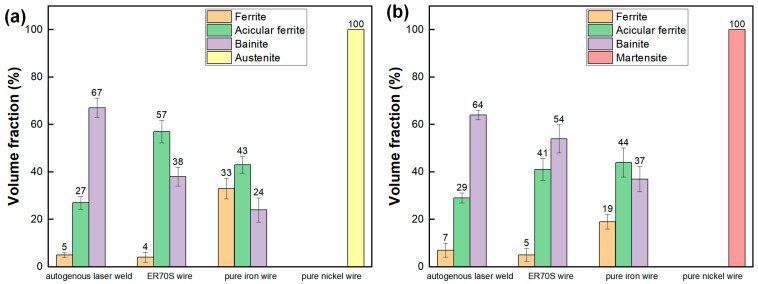
Volume fractions of the various microconstituents observed within the fusion zones of the different welds: (**a**) upper portion; (**b**) lower portion.

**Figure 7:**
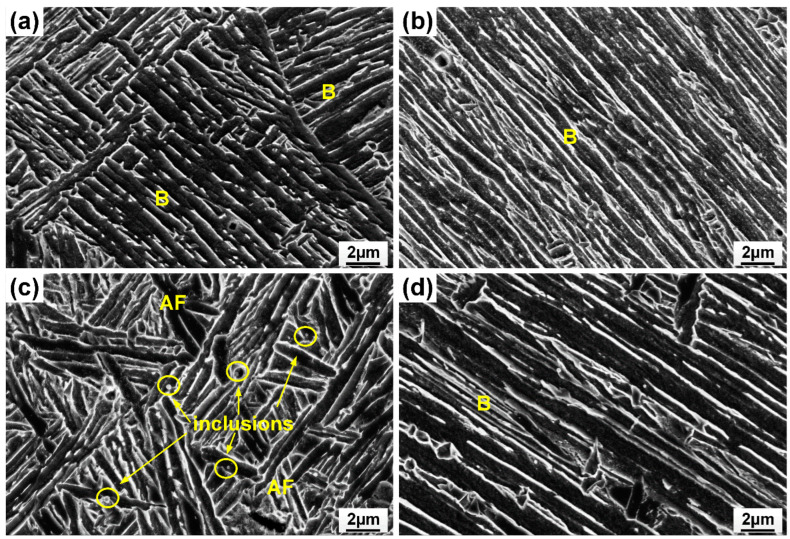
SEM images confirming the presence of bainite (B), acicular ferrite (AF) and non-metallic inclusions in (**a**) upper portion of the autogenous laser weld; (**b**) lower portion of the autogenous laser weld; (**c**) upper portion of the weld with ER70S wire; (**d**) lower portion of the weld with ER70S wire.

Figure 5 shows the microstructure of the weld metal with different filler material. Meanwhile, the volume fraction of microconstituents in the fusion zone is summarized in Figure 6. Resulting from fast cooling, the autogenous laser weld had a bainite-dominated microstructure (with a bainite fraction of 67%), as presented in Figure 5a,b. The introduction of ER70S filler wire during the laser welding process resulted in an increased formation of AF within the weld metal. (Figure 5c,d), which is generally considered the preferred microstructure in pipeline welds due to the high toughness, high strength, and ductility resulting in the fine interlocking grain structure [15]. Specifically designed for carbon steels, ER70S wire can introduce non-metallic inclusions like Si-, Ti- or Mn-based oxides into the weld metal, which act as favorable nucleation sites for forming intragranular acicular ferrite [16,17]. High-magnification SEM images in Figure 7 confirm the presence of bainite and AF in the FZ of the autogenous and ER70S-wire-fed laser welds. Non-metallic inclusions were also found in the ER70S-wire-fed laser weld, as shown in Figure 7c.

As shown in Figure 5e,f, the addition of pure iron wire diluted the alloying elements in the FZ, and led to the formation of more polygonal and coarse-grained ferrite in the fusion zone. Compared to low-temperature transformation products, these constituents will reduce the hardness and strength of the weld metal. However, coarse-grained ferrite may deteriorate low-temperature toughness due to the decreased density of grain boundaries that promote crack deviation during brittle fracture [18]. A possible approach to mitigate the formation of coarse-grained ferrite may be to utilize a wobble laser, which is able to effectively refine the grain structure and improve the intermixing of elements in the molten pool [19,20,21].

Figure 5g shows that a high Ni fraction (38%) in the upper portion of the weld with pure nickel wire led to the formation of austenite, as Ni is a strong austenite stabilizer in steels. Columnar grains, characteristic of rapid solidification, were observed near the fusion line. These grains exhibited a distinct growth pattern, extending along the direction of the temperature gradient during solidification. In contrast, equiaxed grains formed near the weld centerline, due to the decreased temperature gradient in this region. For the lower portion of the FZ, the presence of 12% nickel was not sufficient to form austenite, but would still lower the austenite to ferrite transformation temperature, and led to the formation of lath martensite (LM). One can expect a considerable difference in the local mechanical properties between different portions of the FZ when using nickel filler wire.

**Figure 8:**
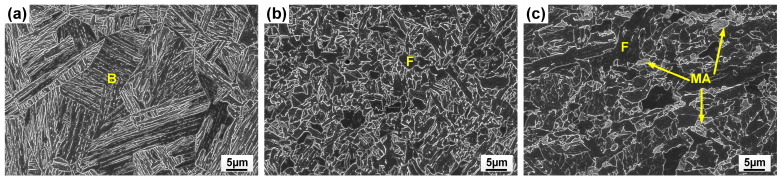
SEM images showing bainite (B), ferrite (F) and martensite–austenite (MA) islands in (**a**) coarse-grained heat-affected zone; (**b**) fine-grained heat-affected zone; (**c**) inter-critical heat-affected zone.

**Figure 9:**
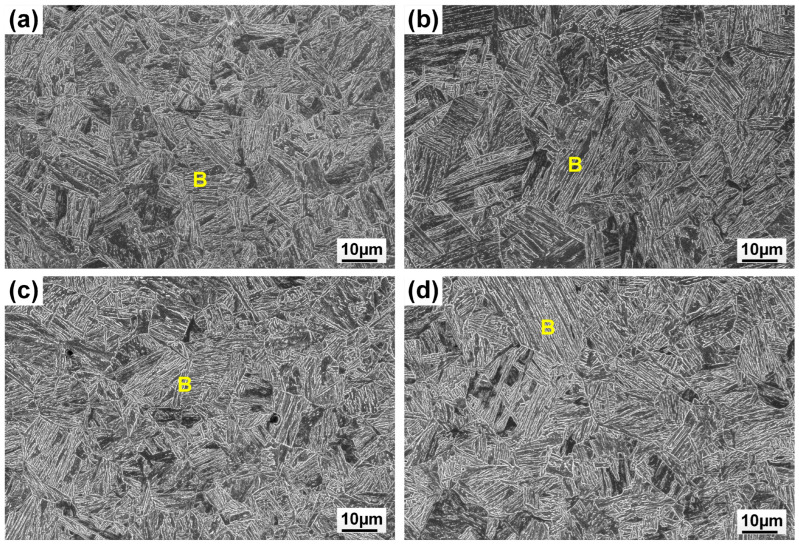
SEM images showing bainite (B) in coarse-grained heat-affected zone: (**a**) autogenous laser weld; (**b**) the weld with ER70S wire; (**c**) the weld with pure iron wire; (**d**) the weld with pure nickel wire.

The subzones of HAZ exhibited varying peak temperatures and cooling rates, leading to different microstructures, as shown in Figure 8. The coarse-grained (CG)HAZ adjunct to the fusion line had a bainitic microstructure resulting from a high peak temperature. Meanwhile, the fine-grained (FG)HAZ, which experienced a low thermal cycle, was composed of equiaxed ferrite. Martensite–austenite islands were found in the inter-critical (IC)HAZ due to the partial re-austenization. Among the subzones of HAZ, CGHAZ is usually considered as a weak point due to the hard and brittle microstructure. As the linear heat input remained the same for all the welding processes with different wires, no notable variation was found in the HAZ microstructure when feeding varying wires, as the CGHAZ microstructures show in Figure 9.

### 3.3. Microhardness

**Figure 10:**
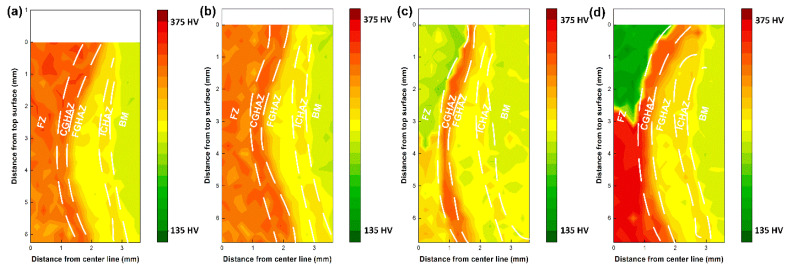
Hardness mapping results, covering the fusion zone (FZ), coarse-grained heat-affected zone (CGHAZ), fine-grained heat-affected zone (FGHAZ), inter-critical heat-affected zone (ICHAZ) and base metal (BM): (**a**) autogenous laser weld; (**b**) laser weld with ER70S wire; (**c**) laser weld with pure iron wire; (**d**) laser weld with nickel wire.

**Figure 11:**
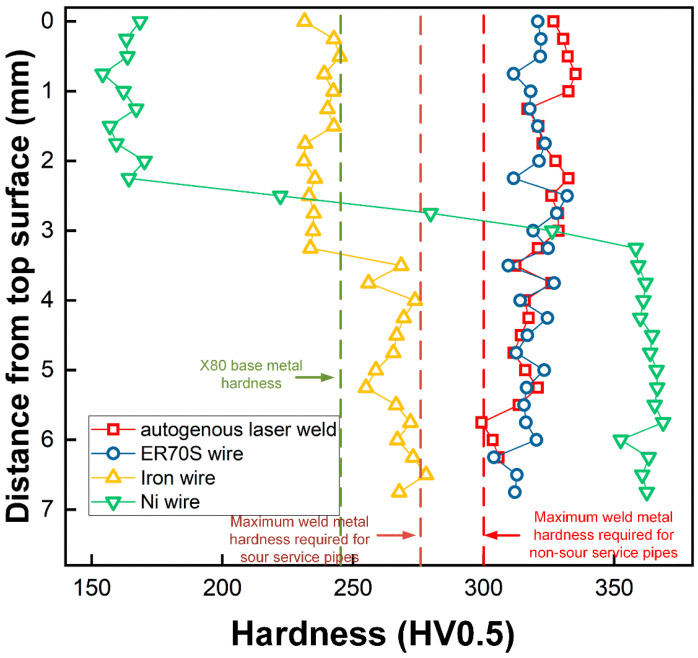
FZ hardness distribution across the thickness of the welds.

The hardness maps and weld metal hardness distribution are shown in Figure 10 and Figure 11, respectively. Meanwhile, the average hardness values in different locations of the welds are also summarized in Table 5. Compared to the autogenous laser weld, the use of ER70S wire resulted in a minor decrease in the hardness of upper portion of FZ, and both the autogenous and ER70S-wire-fed laser welds exhibited a hardness higher than the maximum value required by API (American Petroleum Institute) standards, which is 275 HV for sour service pipes and 300 HV for non-sour service pipes with an X80 strength grade and a thickness greater than 9 mm. It is important to highlight that preheating was not employed in this study to prioritize high productivity, and the results with the highest cooling rates are an extreme case. However, preheating is a recognized strategy in the pipeline industry to mitigate issues with high fusion zone hardness.

As shown in Figure 10c,d, uneven hardness distribution across the thickness of the welds was found in the welds with iron and nickel wire addition, due to the inhomogeneous microstructure. Introducing pure iron wire into the FZ reduced the weld metal hardness from an average of 328 to 237 HV in the upper region and an average from 314 to 267 HV in the lower region, due to the formation of more ferrite. The reduced carbon equivalent caused by the dilution of the elements could also contribute to the decreased weld metal hardness. In comparison with other welds, the weld with iron wire exhibited a hardness closer to that of the X80 base metal and did not exceed the maximum value limited by the API standard. The upper portion of the weld metal with nickel wire had an average hardness of 163 HV, which is 165 HV lower than that of the autogenous weld, due to the formation of soft austenite. However, the presence of martensite in the lower portion led to a much higher hardness of 362 HV (which is 52 HV higher than that of the autogenous laser weld).

Although the wire chemistry significantly affected the fusion zone hardness, the HAZ of these welds exhibited similar hardness due to the same thermal cycle, with the coarse-grained (CG) HAZ presenting a higher hardness than the fine-grained (FG) and inter-critical (IC) HAZs due to a faster cooling. The average CGHAZ hardness was measured to be (332 ± 3 HV), (331 ± 2 HV), (330 ± 2 HV) and (333 ± 3 HV) for the autogenous, ER70S-, iron- and nickel-wire-fed laser welds.

### 3.4. Yield Strength

**Figure 12:**
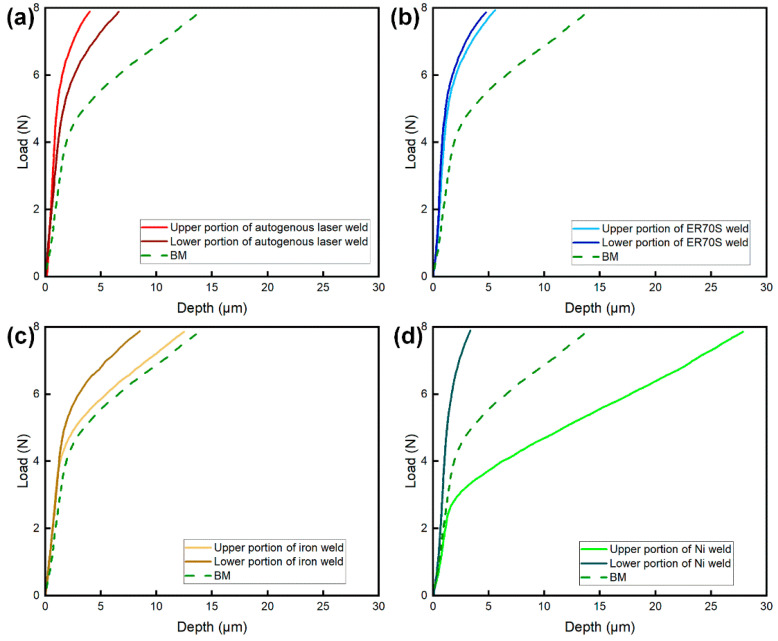
Load–depth curves of the welds obtained through micro-indentation: (**a**) autogenous laser weld; (**b**) the weld with ER70S wire; (**c**) the weld with pure iron wire; (**d**) the weld with pure nickel wire.

**Figure 13:**
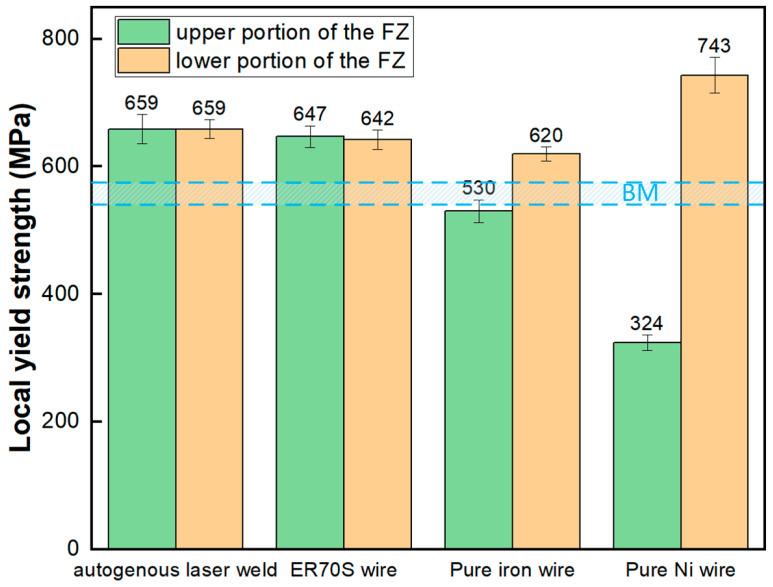
Local yield strength of the weld metal with different wire chemistry.

Figure 12 and Figure 13 present the load–depth curves and yield strength of the welds tested by the micro-indentation technique, respectively. The shallower indentation depths observed in the autogenous (Figure 12a) and ER70S-wire-fed (Figure 12b) laser welds indicated that these two welds were harder than the base metal, with yield strength higher than that of the X80 pipe (556 ± 12 MPa), due to the existence of AF and bainite. The feeding of an iron wire reduced the weld metal yield strength, which can be attributed to the formation of blocky ferrite due to the dilution of alloying elements. As shown in Figure 12d, the addition of nickel wire led to a low yield strength (324 ± 12 MPa) in the upper portion of the weld due to the austenitic microstructure, and a remarkably high strength (743 ± 28 MPa) in the lower portion resulting from the fully martensitic microstructure.

The hardness and yield strength difference in different locations of the FZ, together with the high sensitivity of hot cracking, indicates that with a high wire feed rate, pure nickel wire is not suitable for the welding of pipeline steels. However, it still provides a simple and useful way for quantifying the filler material dilution and extent of mixing promoted by the beam within the fusion zone.

## 4. Conclusions

The findings of this research highlight the influence of wire chemistry and associated elemental dilution on the microstructural evolution and local mechanical properties of X80 HSLA steel laser root welds. Specifically, the study demonstrates the following:

(1) Specifically designed for the welding of low alloyed steels, ER70S filler metal promoted a more desired acicular ferrite microstructure in the laser weld without sacrificing the strength, compared to the autogenous laser weld. Although the filler material was not uniformly distributed along the thickness direction, the local mechanical properties did not differ significantly.
(2) The addition of pure iron wire offers a solution to reduce the weld metal hardness in an X80 laser weld, where the maximum hardness is restricted to not exceed 275 HV in some industrial standards. Despite a 30 HV difference being found between upper and lower portions of the weld metal, hardness and yield strength remained close to the base material. The strategy of diluting alloying elements in the weld metal provides a solution to meet the hardness requirement, before a standard can be developed for laser welding of pipeline steel, or a commercial wire can be specifically modified for the fast-cooling laser welding process. The cracking sensitivity and uneven local mechanical properties make feeding Ni wire not suitable for laser welding of X80 steel; however, it provides a clear tracer element in the case of weld metal flow studies.

## Figures and Tables

**Table 1:** Chemistry of the X80 steel base material.

Element	C	Si	Mn	Ti	Cu	Ni	Nb	Mo	Fe	*P_cm_*
Content (wt.%)	0.051	0.18	1.73	0.01	0.12	0.13	0.04	0.21	Bal.	0.166

**Table 2:** Chemistry of the ER70S-6 steel filler wire.

Element	C	Si	Mn	Ti	Cu	Ni	Mo	Al	Zr	Fe	*P_cm_*
Content (wt.%)	0.07	0.94	1.58	0.01	0.17	0.01	0.01	0.01	0.01	Bal.	0.190

**Table 3:** Wire-fed laser welding parameters.

Sample	Laser Power *P* (kW)	Wire Feed Rate *WFR* (m/min)	Welding Speed *v* (m/min)	Wire Type	Wire Diameter *d* (mm)	Defocused Distance *D_f_* (mm)
1	8	-	1.0	-	-	−3
2	8	6	1.0	ER70S-6	0.9	−3
3	8	6	1.0	Pure iron (99.95%)	0.9	−3
4	8	6	1.0	Pure nickel (99%)	0.9	−3

**Table 4:** Chemical composition in the FZ, obtained through EDS (wt.%).

	Mn	Si	Ni
Autogenous laser weld, upper portion	1.79	0.27	0.13
Autogenous laser weld, lower portion	1.72	0.27	0.13
Laser weld with ER70S wire, upper portion	1.74	0.48	0.09
Laser weld with ER70S wire, lower portion	1.72	0.34	0.15
Laser weld with pure iron wire, upper portion	1.24	0.19	0.10
Laser weld with pure iron wire, lower portion	1.30	0.20	0.12
Laser weld with pure nickel wire, upper portion	1.14	0.22	37.78
Laser weld with pure nickel wire, lower portion	1.56	0.26	11.53

**Table 5:** Average hardness values in different locations of the joints.

	Upper Fusion Zone	Lower Fusion Zone	Base Metal
Autogenous laser weld	328 ± 1 HV	314 ± 2 HV	-
Laser weld with ER70S wire	321 ± 1 HV	317 ± 2 HV	-
Laser weld with pure iron wire	237 ± 1 HV	267 ± 2 HV	-
Laser weld with pure nickel wire	163 ± 1 HV	362 ± 1 HV	-
X80 base metal	-	-	246 ± 1 HV

## Data Availability

The original contributions presented in the study are included in the article, further inquiries can be directed to the corresponding author.
